# Psychedelic-assisted therapy - supposedly paradigm-shifting research with poor attempts at hypotheses falsifying and questionable ethics

**DOI:** 10.17179/excli2024-8023

**Published:** 2024-12-02

**Authors:** Bor Luen Tang

**Affiliations:** 1 Department of Biochemistry, Yong Loo Lin School of Medicine, National University Health System, National University of Singapore, Singapore

## ⁯⁯⁯

The drive for therapeutic uses for psychedelics appears to be gaining momentum, particularly for treatment-resistant depression and post-traumatic stress disorder (PTSD), with several successful phase II and III trials meeting their respective primary outcomes. The Australian Therapeutic Goods Administration has indeed permitted the prescription of psilocybin and N-methyl-1-(3,4-methylenedioxyphenyl)propan-2-amine (MDMA) as unapproved drugs under the Approved Prescriber provisions of the Special Access Scheme, which allows the use psilocybin for intractable depression and MDMA for PTSD as part of a psychedelic-assisted psychotherapy regimen (Haridy, 2023[3]). The much-anticipated approval of MDMA for the treatment of PTSD (MDMA-assisted therapy (MDMA-AP)) in the United States has, however, been recently rejected by the U.S. FDA (Reardon, 2024[10]). This decision is disappointing for patients, clinicians and others who are advocates for the therapy, but perhaps unsurprising from a scientific point of view. U.S. FDA's decision was associated with two Phase III clinical trials for PTSD, MAPP1 and MAPP2, conducted by a consortium funded by Lykos Therapeutics (Mitchell et al., 2021[4], 2023[5]). The trial results appeared rather positive, with MDMA inducing significant attenuation in Clinician-Administered PTSD Scale for DSM-5 score compared with the placebo control arm, or Lykos-specific non-assisted psychotherapy (LSNAP), and significantly decreased Sheehan Disability Scale total score. Predictably, participants receiving MDMA exhibited more common treatment-emergent adverse events (including muscle stiffness, appetite loss, bruxism, hyperhidrosis and fatigue) than LSNAP alone, as well as increased occurrence of event of psychiatric concern, including restlessness and insomnia. However, a report from the Institute for Clinical and Economic Review (ICER) painted a much darker picture:

“*Because of the effects of MDMA, the trials were, essentially, unblinded with nearly all patients who received MDMA correctly identifying that they were in the MDMA arm of the trials. This would always raise concerns about bias, but these concerns are particularly heightened as we heard from multiple experts about the very strong prior beliefs of those involved in the trials (as investigators, therapists, and patients) about the benefits of MDMA-AP. Concerns have been raised by some that therapists encouraged favorable reports by patients and discouraged negative reports by patients including discouraging reports of substantial harms, potentially biasing the recording of benefits and harms*^”^ (Mustafa et al., 2024[7]).

Clinical trials are scientific experiments and should thus follow epistemic and ethical norms for scientific enquiry. The Lykos MDMA-AP trials are clearly problematic in both these regards. Contemporary research on anti-depressants and psychiatric interventions, together with its successes and limitations, represents the current Khunian (Bird, 2022[2]) paradigm. The hope, and thus the supposed paradigm shift in research, is that psychedelics will significantly synergize with, or at least add to, any benefits of psychotherapy, and do so without worrisome adverse effects. To some, this has to happen, or the entire psychedelics driven therapeutic approach would have failed to deliver and the entire enterprise behind the effort would collapse. Such a pressure for positive results can affect human trials at two different levels. Firstly, at the level of those organizing and conducting the trials, and secondly the trial participants. The MDMA-AP for PTSD trials were organized and sponsored by ardent advocates for the use of psychedelics in medicine. The main trial sponsor and organizer, Lykos Therapeutics, is a commercial spin off from the non-profit organization Multidisciplinary Association for Psychedelic Studies (MAPS), whose stated aim is to end psychedelic prohibition. This sense of mission goes beyond the normal interest of commercial companies for profit. As such, there would be significant tendency for hype and bias to creep into trial organization and logistics, as well as data processing and reporting. In the first place, MDMA-AP is made complicated by the presence of the psychotherapist, who could potentially influence the participant's response both in terms of the primary psychiatric indicators, as well as the latter's report of adverse responses. Matters are made complicated even further by the fact that some of the psychotherapists and a good number of trial participants have previously been exposed to MDMA. Many, if not most of them, might faithfully wish for the therapy to be approved.

The hypothetico-deductive model or method (Nola, 2007[8]) is a common approach in scientific research. In the case of psychedelics-assisted therapy (PAT), the general hypothesis would be for the psychedelics to enhance (either additively or synergistically) the effects of psychotherapy in a positive manner, with the corresponding null hypothesis being that the former does not confer any additional benefit. To optimally perform hypothesis-testing and to falsify it, two conditions must be met. Firstly, those administered with psychedelics should be compared to a placebo group, with the individuals randomized for either group. Secondly, whether a participant receives a dose of psychedelic or placebo should be unseen or blind to both the participant and the supervising psychotherapist. However, because the psychotropic effect of psychedelics is so strong, it is very difficult to achieve any true blinding. This is particularly so if either participant or therapist (or both) have used psychedelics, for whom an episode of psychedelic response would be unmistakably familiar. Hypothesis falsifying in the Popperian (Popper, 1959[9]) sense would thus be very difficult, if not impossible. Inadequacy of blinding would already lead to bias, and even more so if either participant or therapist (or both) have any vested interest for PAT to be approved.

Hype and bias notwithstanding, anecdotal evidence presented in the ICER report has also made it clear that there is poor ethical stringency in conducting research. The attending psychotherapists are for all intents and purposes part of the research team. If these researchers believed that MDMA should be approved, they would simply have a biased mindset. However, if they had gone further to influence (or attempt to influence) the participants' responses, their conduct in administering research becomes decidedly aberrant. If the participants are influenced to a point whereby positive effects are exaggerated while negative effects are downplayed, the results of the trial would have essentially been falsified. Some might argue that such inaccuracies are common in all qualitative or semi-qualitative assessments, but falsification is unmistakably a core offence of research misconduct as stipulated by the US Office of Research Integrity (https://ori.hhs.gov/definition-research-misconduct). Furthermore, if the result-collating and interpreting researchers/authors are aware of such potential discrepancies in their raw data, yet presented these nonetheless in publications, they are equally guilty of promoting the falsifications. 

The classic principles of biomedical ethics (Beauchamp and Childress, 2019[1]) include beneficence, non-maleficence, autonomy and justice. Approval of drug indications should balance between risks and benefits. If knowledge of preponderance of adverse events is suppressed while only positive effects are highlighted, the principle of non-maleficence would have been violated, with any apparent beneficence thus distorted. This point might be more acute for some psychedelics than others. MDMA, for example, has been shown to be neurotoxic in animal models and induced neurological and cognitive alterations in humans (Montgomery and Roberts, 2022[6]). Psilocybin, on the other hand, has been shown to be neuroprotective. Coercing or otherwise unduly influencing trial participants violates their autonomy, and falsified trial results served nothing but injustice to patients, clinicians and all other stakeholders. It takes no sophistication in analysis to see that MDMA-AP's research inadequacies are potentially in violation of all the major biomedical principles and if these inadequacies are not eliminated or overcome, they would render the research ethically untenable. If these inadequate practices also plague other PATs, the entire field could eventually become discredited. 

To move forward as a field of research, PAT trials need to fully conform with the gold standards of stringency and integrity, difficult as it may be. To do so, at least two broad classes of issues, the first technical and the other organizational, need to be resolved. One key technical issue is the difficulty in blinding, as sensual perception of a psychedelic experience is simply too strong. There are ways of getting around this problem, such as determining efficacy using dose-response regimens, subperceptual microdosing or a psychedelic trip effect-mimicking control. None of these are ideal solutions and all are much less straightforward to administer in principle. Perhaps the most important reservation on psychedelics concerns adverse events and effects, which could continue to affect the participant long after the initial hallucinogenic trip. How the hallucinogenic trip and other adverse effects associated with it could complicate the psychiatric condition being treated is unclear and requires further work. While it could be argued that the psychedelic trip is important if not critical for the treatment potency of psychedelics, recent findings suggest that the hallucinogenic and the antidepressant effects of psychedelics could potentially be decoupled if the right compounds with altered binding to 5-hydroxytryptamine receptor 2A (5-HT2A) and Tropomyosin receptor kinase B (TRKB) are eventually developed. If antidepressant effects of such new compounds are sufficient, there is no reason to go back to the original compound. 

Administratively, it is crucial to ensure that therapists and trial participants are neutral (or even moderately skeptical) in terms of the therapeutic efficacy of psychedelics. At the very least, they should not be strong advocates for such therapy and should ideally have no previous exposure to psychedelics. Such restrictions might greatly increase the difficulty in participant recruitment but would be necessary to reduce bias. Some have argued that the rejection of MDMA-AP could be due largely to problems that are Lykos-specific, including employment of therapists that are incompetent in terms of research ethics. There are attempts in experimental therapy with psychedelics without accompanying psychotherapy. Although the risk on participants appears to be higher, this will not incur added ethical issues associated with the therapists. What might be more of a Lykos-specific issue is the apparent conflicts of interest given Lykos' declared intention of marketing MDMA for therapy if approved, as individuals with executive positions in Lykos and MAPS were fully involved in the trials and are coauthors on the published papers. Although this involvement is no more than how drug companies are usually involved in drug development and clinical trials, the strong personal enthusiasm for the drug in question beyond a basic quest for profit would add to the problem. It would be better for MDMA based trials to be organized by more neutral parties or organizations, such as a federal agency. At the very least, there should be a neutral and authoritative third party to monitor or scrutinize all protocols, proceedings, results and other relevant aspects of the trials. 

The path to bring a new therapy to the clinic is often fraught with countercurrents of over-enthusiasm and heightened skepticism. It would only be prudent to ensure that the very basics of science and ethics are adequately covered in the research. In the case of PAT, precisely because of its billing as paradigm-shifting research and the difficulty in falsifying hypothesis, the research must maintain impeccable ethical standards. 

## Declaration

### Acknowledgments

The author is grateful to the reviewer whose constructive comments improved the manuscript.

### Declarations

The work receives no funding. The author declares no conflict of interest. No artificial intelligence program was used to assist in writing any part of the manuscript.
